# The Malay version of the Early Childhood Oral Health Impact Scale (Malay-ECOHIS) – assessing validity and reliability

**DOI:** 10.1186/s12955-015-0386-2

**Published:** 2015-11-25

**Authors:** Azlina N. Hashim, Zamros Y. M. Yusof, Rashidah Esa

**Affiliations:** Department of Community Oral Health and Clinical Prevention, Faculty of Dentistry, University of Malaya, 50603 Kuala Lumpur, Malaysia

**Keywords:** Malaysia, Oral health, Preschool children, Quality of life

## Abstract

**Background:**

The Early Childhood Oral Health Impact Scale (ECOHIS) is used to assess oral impacts on the quality of life of preschool aged children and their families. The objective of this study was to perform a cross-cultural adaptation of the ECOHIS into Malay and assess its psychometric properties.

**Methods:**

The cross-cultural adaptation of ECOHIS into Malay comprised of translating the ECOHIS into the Malay language (Malay-ECOHIS) by experts followed by face validation of the Malay-ECOHIS by a group of mothers. The Malay-ECOHIS was back translated into English and this was compared with the original ECOHIS. Minor changes were made to the Malay-ECOHIS before it was finalised. The Malay-ECOHIS’ psychometric properties were assessed in terms of *construct, convergen*t and *discriminant* validity as well as *internal* and *test-retest reliability* based on two separate studies involving 127 parents of 4–6 year old preschool children followed by oral examinations of 860 preschool children from 25 kindergartens from two districts in Selangor state, Malaysia. Non-parametric statistics were used to assess the relationships between the Malay-ECOHIS and the subjective and clinical outcome measures.

**Results:**

The Cronbach’s alpha was 0.83 and the weighted Kappa was 0.95 (intraclass correlation = 0.94). The Malay-ECOHIS demonstrated significant associations with different subjective and normative measures, i.e. levels of oral health satisfaction, perceived oral health status, perceived oral health need, toothache experience, pattern of dental attendance, and caries status of preschool children. These significant associations supported *its construct, convergent* and *discriminant* validity as well as *internal* and *test-retest* reliability.

**Conclusion:**

This study showed that the Malay-ECOHIS is a valid and reliable instrument to assess the negative impacts of oral disorders/conditions on the quality of life of 4–6 year old preschool children and their families in Malaysia.

## Background

Oral health related quality of life (OHRQoL) indicators have been developed to assess the impact of oral conditions on daily life [1]. In young children, assessing oral impacts on daily performances is important because poor oral health can affect not only their future dentition, but also their growth, weight, social life, self-esteem and school performance [2, 3]. In addition, as they depend a lot on their parents, dental problems in young children often have consequences on their parents, caregivers, and siblings who live with the child [4, 5]. In severe cases where the child’s oral impacts persist for years, their effect on the child may last until adulthood [6].

In young children, assessing oral impacts on daily performances requires a special approach. This is because young children often have cognitive limitations in recalling past events. Their memories may be unreliable and they are unable to express themselves fully. Thus, parent’s input to validate such findings is essential [3, 7]. In addition, children’s oral health problems do not exist in isolation but often affect other family members in real life situations [5]. Thus, when measuring young children’s OHRQoL, parental input on children’s oral health problems and their impacts on the child’s wellbeing, their siblings and the family dynamics is important [8].

Taking into account the various limitations in assessing OHRQoL in young children, the Early Childhood Oral Health Impact Scale (ECOHIS) was developed to overcome such limitations [9]. The primary objective of ECOHIS is to measure young children’s oral impacts on daily life including those of family members. The scale has been validated on 3–5 year old children and their families in English in the USA. It has been translated into several versions, i.e. French, Chinese, Farsi, Turkish, Spanish, Lithuanian, Bosnia-Herzegovina and Brazilian [10–17]. Overall, the ECOHIS showed high degree of success in the assessment of oral impacts in young children with medical conditions [18, 19], traumatic dental injuries and malocclusions [20] and those with early childhood caries [21]. It has also been used to assess young children’s OHRQoL following treatment under general anaesthesia [22]. In the community, the use of the scale would allow for the development of effective oral health programmes and services because it permits assessment of young children’s perceived needs, levels of care required and treatment strategy effectiveness [8, 23, 24].

The aim of the present study was to perform a cross-cultural adaptation of the English ECOHIS into Malay followed by psychometric analysis of the Malay-ECOHIS.

## Methods

The development of the Malay-ECOHIS involved two phases. The first phase involved the cultural adaptation of the English ECOHIS into Malay [25]. The second phase involved psychometric validations of the newly developed Malay-ECOHIS [26, 27].

In the cross-cultural adaptation phase, the English ECOHIS was first translated into the Malay language by a team of independent translators consisted of a psychologist, a peadodontist, dental public health specialists and experts in quality of life assessment. The experts were also proficient in both Malay and English languages. Next, the experts met together to analyse the content and wordings of the translations. The objective was to ensure that conceptual and item equivalence between the original ECOHIS and its Malay versions were maintained throughout the process [28]. Conceptual equivalence is achieved when answers to the same questions reflect the same concept in the original ECOHIS and its Malay version respectively, and also the concepts are meaningful in both cultures and languages concerned. Item equivalence is achieved when the meaning of the individual item in both sets of questionnaires is contextually similar. Following the meeting, the experts agreed on one consensus translation derived from the translations. The consensus translation was called the draft Malay-ECOHIS.

Next, the draft-Malay ECOHIS was tested for face validity on a non-random sample of 20 mothers of 4-6 year old children in a classroom setting at one of the kindergartens supervised by one of the authors (RE) [26]. The time taken to answer the questionnaire was noted. Afterwards, the author (RE) undertook a detailed discussion with the mothers on their understanding of the purpose, instructions, content, wordings, answering options, and general layout of the questionnaire. Based on the mothers’ feedback, a minor adjustment was made to the draft Malay-ECOHIS. The mode of questionnaire administration was self-administered.

Next, the draft Malay-ECOHIS was back translated into English by a language expert from the Department of Languages, University of Malaya who was proficient in both English and Malay languages. Then, the experts reconvened to compare the back translation with the original ECOHIS. After minor modifications, the experts agreed on the back translation of the Malay-ECOHIS. Small changes to the draft Malay-ECOHIS were made accordingly before it was finalised.

The assessment of the Malay-ECOHIS psychometric properties involved 2 studies. In study 1, the Malay-ECOHIS was distributed by one of the authors (NAH) to a convenient sample of 127 parents of 4-6 year old children from two public and one private kindergarten in Kelana Jaya district in the Selangor state. To assess the test-retest reliability, the scale was redistributed to 20 % of the sample after 10 days [9]. In study 2, in order to assess the relationship between the Malay-ECOHIS and clinical outcomes, the scale was distributed by RE and ZY to 860 parents of 4–6 year old preschool children from 25 kindergartens from 2 districts in Selangor state. Oral examinations were undertaken on the children.

### The questionnaire

The Malay-ECOHIS comprised 13 items divided into two main parts, i.e. the child impacts section and the family impacts section. The number of items and sections in the Malay-ECOHIS were similar to the original ECOHIS [9]. In the Malay-ECOHIS, the child impacts section contained four domains, i.e. child symptom (1 item), child function (4 items), child psychology (2 items) and child self-image and social interaction (2 items). The family impacts section contained two domains, i.e. parental distress (2 items) and family function (2 items).

The questionnaire has 5 response options: 0 = never, 1 = hardly ever, 2 = occasionally, 3 = often, 4 = very often, and 5 = don’t know. The different score ranges for each domain were as follows: child symptom, range 0–4; child function, range 0–16; child psychology, range 0–8; child self-image/social interaction, range 0–8; parental distress, range 0–8 and family function, range 0–8. Total score was calculated by simply summing up the response codes of the 13 items. Thus, the overall score ranged between 0 and 52 (0–36 for the child section and 0–16 for the family section). In our study, we dealt with ‘don’t know’ (DK) responses by following the method of data scoring proposed in the original version, where DK responses were recoded as missing values [9]. In cases with up to 2 missing responses in the child section or 1 missing response in the parent section, we ascribed the score for the missing value as the average of the rest of the items for that section [9]. Subsequently, we only excluded cases with more than 2 missing responses in the child section or more than 1 in the family section. When answering the Malay-ECOHIS, parents were asked to consider the lifetime experience of the child with regard to oral problems and related impacts. Higher scores indicate greater oral impacts and poorer OHRQoL and vice versa.

In our study, the inclusion criteria were mothers of children aged between 4 and 6 years with no chronic medical conditions, no long-term medication, no physical or learning disabilities, and accompanied by a Malay-speaking parent who lived with the child most of the time.

### Ethics, consent and permissions

Ethical approval for the study was granted by the Medical Ethics Committee, Faculty of Dentistry, University of Malaya [Reference: DF CO1403/0042(P)]. Permission to conduct the study was obtained from the State Education Department, State Oral Health Division (Selangor), kindergarten teachers and parents of the children.

Information about the study was given to the parents to read. A written consent from the parent was obtained before they answered the questionnaire.

### Data analysis

In this study, psychometric properties of the Malay-ECOHIS were analysed by assessing its internal consistency and test-retest reliability as well as construct, convergent and discriminant validity.

For *internal consistency reliability*, Cronbach’s alpha coefficient, inter-item correlation and corrected item-total correlation were used to assess the degree of homogeneity of the child and family impacts sections. Cronbach’s alpha values ≥ 0.70 were considered acceptable for comparison between groups [29]. The *test-retest reliability* test was carried out to ensure the Malay-ECOHIS would yield consistent scores when administered at two different times [30]. This was determined by the weighted kappa value for categories of Malay-ECOHIS scores and Intraclass Correlation Coefficient (ICC) in a one-way random effect parallel model for the child and family impacts sections. The 95 % confidence interval was estimated. The degree of test-retest reliability was assessed based on the ICC values, i.e. ≤0.40 = weak, 0.41 to 0.60 = moderate, 0.61 to 0.80 = good, and 0.81 to 1.00 = excellent [31]. Arbitrary guidelines characterized kappa value over 0.75 as excellent, 0.40 to 0.75 as fair to good, and below 0.40 as poor [32].

The ability of the Malay-ECOHIS to assess pre-school children’s OHRQoL was assessed by examining the association between Malay ECOHIS scores and a number of subjective variables designed to indicate, both objectively and subjectively, the levels of oral health status and quality of life of the study population.

*Convergent validity* of the Malay-ECOHIS was tested on its ability to measure what it intended to measure [30]. In this study, the Malay-ECOHIS was intended to measure child’s oral impacts which also mirrored levels of child’s oral health status. Consequently, the convergent validity was tested by comparing its relationship with a suitable global oral health rating item on perceived oral health status of the child, i.e. “How do you describe your child’s oral health status?” The underlying hypothesis was that parents who rated their child’s oral health status as poor would score highly on the Malay-ECOHIS.

*Construct validity* of the Malay-ECOHIS was assessed by comparing its relationships with other measures that assess related constructs, i.e. perceived satisfaction on child’s oral health, perceived child’s treatment needs, and presence of toothache. The items used were (1) “How satisfied are you with your child’s teeth/mouth?” (2) “In your opinion, would your child require any dental treatment?” and (3) “How often has your child had pain in their teeth, mouth or jaws?” The hypothesis related to the tests was that preschool children whose oral health was rated as less satisfactory and needed dental treatment and those with pain in their teeth and mouth would experience lower levels of OHRQoL and higher Malay-ECOHIS scores. The impacts of child’s oral health on his/her daily life were also closely related to the impacts on family members [8, 9].

*Discriminant validity* of the Malay-ECOHIS was tested by comparing its relationship with the child’s dental visits due to dental problems and the child’s caries status. The hypothesis behind this was that mothers who often brought their child to the dentist for treatment were more likely to report that their child experienced dental problems. Likewise, children with caries would have significantly higher oral impacts than children with no caries.

In this study, the Malay-ECOHIS scores were skewed. Therefore, non-parametric statistics, i.e. Kruskal-Wallis and Mann Whitney were used to assess relationships between the Malay-ECOHIS and subjective/objective measures [30]. Data distribution was described in terms of mean and median. The SPSS statistical package version 17 was used for data analysis [33]. The level of statistical significance was set at *p* < 0.05.

## Results

The 127 participating parents comprised parents of 64 boys (50.4 %) and 63 girls (49.6 %). The children’s mean age was 5 years old (SD = 0.6). The sample was predominantly Malay (*n* = 124, 97.6 %), 2 Indian (1.6 %), and 1 Chinese (0.8 %) with varying socioeconomic background (Table 1).

**Table 1:** Demographic characteristics of the sample of preschool aged children and their parents (*N* = 127)

Demographic characteristic	Frequency	
	n	%
Child’s characteristics		
Age (years)		
6	61	48.0
5	55	43.3
4	11	8.7
Gender		
Boys	64	50.4
Girls	63	49.6
Race		
Malay	124	97.6
Chinese	1	0.8
Indian	2	1.6
Kindergarten		
Tadika KEMAS Nurul Jamruk (public)	45	35.4
Tadika KEMAS Nur Ar-Rahim (public)	15	11.8
Tadika Cahaya Taqwa (private)	67	52.8
Child’s position in the family		
Eldest child	36	28.3
Second child	35	27.6
Third child	24	18.9
Fourth child	21	16.5
Fifth child	7	5.5
Sixth child	3	2.4
Other	1	0.8
Parent’s characteristics		
Relationship to child		
Mother	79	62.2
Father	48	37.8
Level of education (mother)		
Secondary school	48	37.8
Diploma or equivalent	34	26.8
University	45	35.4
Level of education (father)		
Secondary school	53	41.7
Diploma or equivalent	35	27.6
University	39	30.7
Monthly family income^a^		
RM 1000 – RM 2999 (low)	27	21.3
RM 3000 – RM 4999 (moderate)	58	45.7
> RM 5000 (high)	42	33.0

^a^RM = Ringgit Malaysia (RM1 = USD 0.27 = GBP 0.18)

More children attended private (*n* = 67, 52.8 %) than public kindergartens. More than one-third of the children were the eldest in the family (*n* = 36, 28.3 %) and the mean number of siblings was 3.1 (SD = 1.4). The majority of parents were mothers (62.2 %). More than one-third of mothers (35.4 %) and 30.7 % of fathers had education up to university level. About one-fifth of parents had monthly income of RM1000–2999 (low income level), less than half (45.7 %) had monthly income of RM 3000–4999 (moderate income level) and 33.0 % had monthly income of > RM5000 (high income level).

Table 2 shows the parents’ responses to the Malay-ECOHIS with regard to their child’s oral conditions. In the child impacts section, less than half of children were reported to have suffered from “pain in the teeth, mouth or jaws” (40.9 %). The most frequently reported oral impact was “difficulty in eating some foods” (27.6 %), followed by “difficulty in drinking hot and cold beverages” (18.1 %), “became irritable or frustrated” (17.3 %), and “had trouble sleeping” (15.7 %).

**Table 2:** Malay-ECOHIS responses of parents (*n* = 127)

Impacts	Never		Hardly ever		Occasionally		Often		Very often		Don’t know		Mean (SD)
	n	%	n	%	n	%	n	%	n	%	n	%	
Child impacts													
How often has your child had pain in the teeth, mouth or jaws	75	59.1	9	7.1	41	32.3	1	0.8	0	0	1	0.8	0.75 (0.95)
How often has your child ………… because of dental problems or dental treatments?													
had difficulty drinking hot or cold beverages	104	81.9	7	5.5	15	11.8	0	0	0	0	1	0.8	0.29 (0.67)
had difficulty eating some foods	92	72.4	13	10.2	20	15.7	2	1.6	0	0	0	0	0.46 (0.81)
had difficulty pronouncing any words	114	89.8	5	3.9	6	4.7	0	0	1	0.8	1	0.8	0.17 (0.58)
missed preschool, day care or school	124	97.6	1	0.8	0	0	0	0	0	0	2	1.6	0.01 (0.09)
had trouble sleeping	107	84.3	8	6.3	12	9.4	0	0	0	0	0	0	0.25 (0.62)
been irritable or frustrated	105	82.7	10	7.9	10	7.9	0	0	0	0	2	1.6	0.24 (0.59)
avoided smiling or laughing	114	89.8	5	3.9	8	6.3	0	0	0	0	0	0	0.17 (0.52)
avoided talking	118	92.9	4	3.1	4	3.1	0	0	0	0	1	0.8	0.10 (0.39)
Family impacts													
How often have you or another family member ………… because of your child’s dental problems or treatment?													
been upset	86	67.7	25	19.7	12	9.4	3	2.4	0	0	1	0.8	0.46 (0.77)
felt guilty	79	62.2	26	20.5	15	11.8	5	3.9	0	0	2	1.6	0.57 (0.86)
taken time off from work	112	88.2	11	8.7	3	2.4	0	0	0	0	1	0.8	0.13 (0.41)
How often has your child had dental problems or dental treatments that had a financial impact on your family?	120	94.5	2	1.6	3	2.4	0	0	0	0	2	1.6	0.06 (0.33)

In the family impacts section, the most frequently reported impacts were “parents or family members feeling guilty” (37.8 %) and “felt upset” (32.3 %) as the results of the child’s oral conditions. In the child impacts section, 2 parents chose “Don’t Know” on the items related to “missed preschool, day care or school” and “their child had been irritable or frustrated”. In the family impacts section, 2 parents answered “Don’t Know” on the items “felt guilty” and “financial impact on family”. In this study, the Malay-ECOHIS scores ranged from 0 to 17 in the child impacts section (mean = 2.4, SD = 3.6), and 0–7 in the family impacts section (mean = 1.2, SD = 1.8).

Table 3 shows the inter-item correlation coefficients of the 13-item scores of the Malay-ECOHIS. The inter-item correlation coefficients ranged from 0.005 to 0.807. The weakest correlation was between items “irritation” and “financial impact” with coefficient value of 0.005 while the strongest correlation was between items “smiling” and “talking” with coefficient value of 0.807. Six correlations involving the item “financial” had negative coefficient values.

**Table 3:** The Malay-ECOHIS reliability analysis: inter-item correlation coefficients of the 13 items

	Pain	Drinking	Eating	Pronouncing	Absence	Sleeping	Irritation	Smiling	Talking	Upset	Guilty	Work	Financial
Pain	1.000												
Drinking	.489	1.000											
Eating	.491	.641	1.000										
Pronouncing	.350	.532	.406	1.000									
Absence	.121	.236	.180	.129	1.000								
Sleeping	.455	.467	.332	.185	.115	1.000							
Irritation	.415	.478	.615	.249	−.038	.533	1.000						
Smiling	.219	.292	.546	.122	−.030	.347	.622	1.000					
Talking	.110	.301	.416	.211	−.023	.385	.521	.807	1.000				
Upset	.270	.200	.386	.363	.066	.124	.311	.271	.126	1.000			
Guilty	.373	.280	.323	.330	.049	.263	.392	.113	.084	.763	1.000		
Work	.196	.304	.138	.137	.190	.195	.176	.083	.167	.219	.328	1.000	
Financial	−.001	−.013	−.049	−.060	−.018	−.039	.005	.031	.075	.077	.106	.172	1.000

Table 4 shows the corrected item-total correlation of the 13 items of the Malay-ECOHIS. The corrected item-total correlation values were all positive ranging from 0.04 to 0.68. Of the 13 items, 11 had corrected item-total correlation values above 0.3. The lowest value was related to “financial impact” (0.04) while the highest value was related to “eating” (0.68) and “irritation” (0.68). The Cronbach’s alpha coefficient was 0.83 and the coefficient only increased by 0.01 if the item on financial impact was deleted. For the test-retest reliability, the weighted kappa value was 0.95 and the intraclass correlation coefficient (ICC) was 0.94.

**Table 4:** Reliability analysis: corrected item-total correlation of the 13 items of the Malay-ECOHIS, Cronbach’s alpha coefficient, Intraclass Correlation Coefficient (ICC) and kappa coefficient

	Corrected item-total correlation	Cronbach’s alpha if item deleted
Pain	.55	.81
Drinking	.64	.80
Eating	.68	.80
Pronouncing	.47	.82
Absence	.15	.83
Sleeping	.50	.81
Irritation	.68	.80
Smiling	.49	.82
Talking	.45	.82
Upset	.51	.81
Guilty	.54	.81
Work	.32	.83
Financial	.04	.84
Cronbach’s alpha coefficient	0.83	
Intraclass Correlation Coefficient (ICC)	0.94	
Weighted kappa value	0.95	

Table 5 shows the results of the construct, convergent and discriminant validity tests of the Malay-ECOHIS. There was a trend of increasing Malay-ECOHIS scores from parents who were “very satisfied” to those who were “very unsatisfied” with their child’s teeth/mouth (*p* < 0.001). Similar trend was observed on parents who perceived their child’s oral health status as “excellent” to those who perceived their child’s oral health status as “poor” (*p* < 0.001).

**Table 5:** Construct, convergent and discriminant validity tests for the Malay-ECOHIS - associations between Malay-ECOHIS and subjective outcome variables (*n* = 123)

Variables	n	Mean	(SD)	ECOHIS score (Quartiles)	P*
Perceived satisfaction on child’s oral health					
Very satisfied	8	0.88	(2.47)	(0.0, 0.0, 0.0)	<0.001
Satisfied	56	2.21	(3.72)	(0.0, 0.0, 3.8)	
Moderate	37	4.68	(4.47)	(0.0, 4.0, 8.0)	
Not satisfied	18	5.22	(4.75)	(1.8, 4.0, 8.3)	
Very unsatisfied	4	11.25	(7.41)	(5.3, 9.5, 19.0)	
Perceived child’s oral health status					
Excellent	7	1.00	(2.65)	(0.0, 0.0, 0.0)	<0.001
Very good	19	1.42	(3.82)	(0.0, 0.0, 0.0)	
Good	50	3.16	(4.01)	(0.0, 1.5, 5.0)	
Moderate	40	4.65	(4.59)	(1.0, 4.0, 7.8)	
Poor	7	9.29	(6.18)	(5.0, 6.0, 13.0)	
Perceived child’s oral health need					
Yes	94	4.57	(4.80)	(0.0, 4.0, 7.0)	<0.001
No	21	0.48	(1.44)	(0.0, 0.0, 0.0)	
Don’t know	8	0.38	(0.74)	(0.0, 0.0, 0.75)	
Child’s experience of toothache					
Very often	1	19.0	(0.00)	(19.0, 19.0, 19.0)	<0.001
Occasionally	48	6.50	(4.68)	(3.0, 5.0, 10.0)	
Never	74	1.51	(2.81)	(0.0, 0.0, 3.0)	
Dental visit due to dentally related problems					
Never	55	1.96	(3.72)	(0.0, 0.0, 3.0)	<0.001
Occasionally when have problems	62	4.92	(4.60)	(1.0, 4.0, 8.0)	
Regularly	6	5.00	(7.18)	(0.8, 2.0, 9.3)	

*Kruskal-Wallis non-parametric statistics

Parents who perceived their child as needing dental treatment had significantly higher Malay-ECOHIS scores than those who perceived their child as not needing dental treatment. Those who were unsure had lowest Malay-ECOHIS scores compared with the other two groups of parents. The trend was statistically significantly (*p* < 0.001).

Parents who reported their child had toothache “very often” had significantly higher Malay-ECOHIS scores than those who reported their child had “occasional” toothache; and those who reported their child had no toothache at all (*p* < 0.001).

Children who never went to see the dentist because of dental problems had significantly lower Malay-ECOHIS scores than children who went to the dentist occasionally; and children who went to the dentist regularly because of dental problems (*p* < 0.001).

Table 6 shows further evidence on discriminant validity of the Malay-ECOHIS. For each of the child impacts section, family impacts section, and the overall score, the mean Malay-ECOHIS scores were significantly higher in children with caries than children without caries. The effect size for each section was small with caries-free children having better OHRQoL than children with caries. Also, there is some suggestion of floor effects for the child impacts section, parent impacts section and the overall scale which had 21 % or more scoring 0 on each section respectively.

**Table 6:** Discriminant validity of Malay ECOHIS through comparison of mean Malay-ECOHIS scores and respective sub-scales by caries status

M-ECOHIS	Caries	Caries-free	p-value*	Cohen’s d^a^	% Floor	% Ceiling
	Mean (SD)	Mean (SD)			(0 score)	(max score)
Child impact section	13.1 (4.00)	12.17 (4.78)	<0.001	0.21	27.0	0.1
Family impact section	5.35 (2.01)	4.85 (2.18)	<0.001	0.24	58.4	0.2
Overall score	18.44 (5.39)	17.00 (6.46)	<0.001	0.24	21.6	0

*SD* standard deviation

*Mann-Whitney statistics

^a^0.21 < d < 0.24 = small effect size respectively

## Discussion

In this study, the original English ECOHIS was cross-culturally adapted into Malay by following a standard published procedure [25, 26]. Overall, based on the validity and reliability tests analyses, the 13-item Malay-ECOHIS has been proven to be valid and reliable for use by parents of 4–6 year old pre-school children in Malaysia to assess children’s oral impacts on quality of life and their family. The questionnaire’s mode of administration was self-administered.

In the content validation analysis, the experts had unanimously agreed that the forward and back translations of the Malay-ECOHIS satisfied the content and item equivalence requirements between the English ECOHIS and its Malay version [28]. The back translation was found to be closely similar to the original ECOHIS with a slight adjustment made to the Malay version.

In the face validation test analysis, the utility of the Malay-ECOHIS as a self-administered questionnaire had been verified and agreed upon by a group of mothers through discussion after they had answered the questionnaire. Their input was taken into consideration in the final draft of the Malay-ECOHIS. In this pre-test, only mothers were involved as most mothers were present during the pre-test and volunteered for the study. None of the fathers whom we approached were willing to volunteer. In the Asian culture, mothers play the dominant role in raising the child and are more willing to participate in activities concerning the child’s welfare. Also, the majority of mothers are home makers and thus have more time with their child. As such, their input and understanding of the scale was important. As the maternal influence in the child upbringing is foremost, we felt the absence of fathers in the pre-test would not compromise the validity of the scale in a significant way.

Through content and face validation test analyses, the experts verified that the 13-item Malay-ECOHIS was contextually similar to the original ECOHIS. The mothers commented that the scale was relatively short, easy to understand, relevant, and suitable to assess young children’s oral impacts in the Malaysian setting.

In terms of the layout, the Malay-ECOHIS followed the original ECOHIS in terms of design and presentation. It was interesting to note that the 3 most common impacts reported by parents in the child impacts section were similar to those found in the earlier studies of different cultures and settings [9, 10, 17]. These responses were “pain in the teeth, mouth or jaws”, “difficulty in eating some foods” and “child being irritable or frustrated”. This indicates that the Malay-ECOHIS is comparable to other ECOHIS versions to detect prevalent oral impacts among preschool children across different cultures and settings.

In the internal consistency reliability test analysis in study 1, almost all of the inter-item correlations of the 13 item scores were positive and coefficients ranged from 0.005 to 0.807. None of the values were above the coefficient value of 0.9 indicating that no items were deemed redundant. The items were mostly correlated with one another in a positive manner. Only one item, i.e. ‘financial impact’, had negative inter-item coefficients with six other items in the child impacts section. However, the negative values were extremely small. Further psychometric analysis showed no unfavourable effect of the financial impact item on the scale’s overall performance. Thus, it was decided to keep the financial impact item in the scale as it was deemed relevant to its overall construct. The corrected item-total correlations were all positive and 11 of the 13 correlations were above 0.2 indicating that most of the 13 items correlated well with the total score and the scale overall [30]. Furthermore, the Cronbach’s alpha value was 0.83 indicating that the scale has good internal consistency, higher than the recommended value of 0.70 [34]. The Cronbach’s alpha value did not increase significantly if the financial impact item was deleted. Also, the value was lower than 0.95 indicating that no item was deemed redundant nor overlapped with another in the construct. Other studies on ECOHIS validation also reported Cronbach’s alpha values [10–12, 17].

In the test-retest analysis, the weighted Kappa score was 0.95 and the intraclass correlation coefficient (ICC) value for the total score was 0.94 indicating that the Malay-ECOHIS has excellent levels of agreement. In effect, it shows that the scale has excellent test-retest reliability where it is able to yield consistent scores when administered at two different times [35]. The value of ICC was similar with that reported in the Brazilian study [17] but higher than that reported in the original, Farsi, and Chinese versions of the ECOHIS (0.84, 0.82 and 0.64, respectively) [9, 11, 12].

In the construct, convergent and discriminant vitality test analyses in study 1, only responses from 123 parents were included. Responses from 4 parents were omitted because they either had more than 2 missing responses in the child section or more than 1 missing responses in the parent section [9]. Overall, the analyses showed that the Malay-ECOHIS had excellent validity in the 3 tests, respectively. In the convergent validity test, the Malay-ECOHIS showed significant association with perceived oral health status of the children. This finding was consistent with findings from other studies where parents who perceived their child’s oral health status as poor had significantly higher ECOHIS scores [9, 10, 12, 17]. This finding also supports suggestions that parents can provide valid reports on preschool children’s OHRQoL when these conditions are observable [3, 9].

In the construct validity test, the Malay-ECOHIS showed significant associations with children’s levels of perceived oral health satisfaction, perceived oral health need, and toothache experience. These findings empirically supported the construct validity of the scale.

In the discriminant validity test analysis, the Malay-ECOHIS has been shown to be able to discriminate between children who went to see a dentist regularly because of oral problems and children who did not see a dentist because they had no dental problems. In Malaysia, pre-school children receive oral examination once a year by dental nurses. Those with no dental problem would not need further treatment and thus only seen once a year by the dental nurses. Those with dental problems, e.g. toothache or decayed teeth, are referred to a dentist for further treatment. Thus, in our study those who went to see the dentist very often were the ones with dental problems with relatively higher oral impacts. The discriminant validity of the scale was proven clinically in study 2 when children with caries had significantly higher Malay-ECOHIS scores than those with no caries.

In study 1, 14 subjects (11 %) had DK responses in the child or family impacts section or both. This percentage was similar to the French study [10] but slightly higher than that in the original ECOHIS study (7 %) [9]. In our study, we decided to include subjects with DK responses in the analysis following the criteria and method of data analysis recommended in the original study [9]. This method of data analysis was similar to other validation studies on the ECOHIS [10, 13, 17]. Furthermore, our decision to include subjects with DK responses in the analysis was based on comments by Jokovic et al in which DK response option is considered essential when a person is tasked to assess the oral impacts and quality of life of another individual and they should be included in the analysis. According to Jokovic et al, the presence of DK responses in the answers reflects specific features of the occurrence under assessment and mirrors the construct being examined rather than errors by the respondents [36]. As a result, as many as 10 out of 14 subjects with DK responses in their answers were included in the analysis.

The sample size (*n* = 127) in study 1 may be criticised to be a bit small. However, sample size requirement in studies on psychometric analysis depends predominantly on the number of items in the scale. In general, for scale with 20 items or less, a sample size between 100 and 200 subjects was reasonable [37]. According to quality criteria for measurement properties of health status questionnaires, at least 50 subjects were necessary for an appropriate analysis of construct validity, reproducibility, responsiveness, and a minimum of 100 subjects are required to perform internal consistency analysis [38]. Thus, for the Malay-ECOHIS psychometric analysis with 13 items, the sample size of 127 was considered sufficient for the purpose of testing its psychometric properties.

This study has a few limitations. Only 4–6 year old children were included in the main psychometric analysis although the scale was developed for 1–5 year old children [9]. Variation in age group between this study and other similar studies was mainly due to logistic reasons. In Malaysia, 1–3 year old children is difficult to reach as the majority stay at home while only a minority attend day care centres. As such, there is no meaningful method to capture this age group except during dental visits or at the maternal and child health clinics. However, the number is small and unpredictable. On the other hand, the 4–6 year old group is readily accessible as they attend preschool centres and kindergartens before they enter primary schools. This age group receives various government funded health programmes on yearly basis including annual dental check-up and oral health promotion activities by dental nurses. The use of the scale in this age group has added value in that it may potentially allow assessment of oral health programmes effectiveness particularly against caries as well as to prioritise care, evaluate treatment outcomes and compare preschool children’s oral health over time.

The use of the Malay-ECOHIS was based on parent’s perceptions of their child’s oral conditions and their impacts on the child and family. Consequently, different parents might have different perceptions of their child’s oral health. The period of assessment was also different from child to child based on their age. Some parents were uncertain whether or not to include problems with teething as oral impacts when answering the questionnaire. However, this was explained by the researchers when parents were answering the questionnaire.

## Conclusion

This study has shown that the Malay-ECOHIS is a valid and reliable measure to assess negative impacts of oral conditions on the quality of life of 4–6 year old preschool aged children and their families in Malaysia.

## Abbreviations

ECOHIS: Early childhood oral health impact scale
OHRQoL: Oral health related quality of life
ICC: Intraclass correlation coefficient
